# Musculoskeletal Sequelae of Post-COVID-19 Syndrome: A Systematic Review

**DOI:** 10.3390/diseases13120391

**Published:** 2025-12-03

**Authors:** Claudia Tatiana Zuñiga-Jimenez, Diego Fernando Rojas-Esguerra, Aida Paola Muñoz-Martinez, Diana Carolina Mendoza-Guzman, Jorge Enrique Daza-Arana

**Affiliations:** 1 Physiotherapy Program, Faculty of Health, Universidad Santiago de Cali, Palmira 763532, Colombia; aida.munoz00@usc.edu.co (A.P.M.-M.); diana.mendoza00@usc.edu.co (D.C.M.-G.); jorge.daza01@usc.edu.co (J.E.D.-A.); 2 Health and Movement Research Group, Faculty of Health, Universidad Santiago de Cali, Cali 760001, Colombia; 3 Physiotherapy Program, Faculty of Health Sciences, Universidad de San Buenaventura, Cartagena 130010, Colombia; diego.rojas@usbctg.edu.co; 4 Specialization in Internal Medicine, Faculty of Health, Universidad Santiago de Cali, Cali 760001, Colombia

**Keywords:** long COVID, muscle strength, musculoskeletal pain, sarcopenia, weakness, fatigue

## Abstract

**Background/Objectives:** COVID-19 infection is a respiratory illness that affects multiple body systems, including the musculoskeletal system. In August 2024, Colombia reported 6 million infections and a 2.2% mortality rate related to COVID-19. Post-COVID-19 syndrome (PCS) is a chronic condition occurring after the acute infection, typically characterized by fatigue, weakness, pain, and sarcopenia, impacting the patient’s quality of life (QoL). This systematic review aimed to identify musculoskeletal sequelae, including peripheral muscle strength, fatigue, and QoL, in patients with PCS. **Methods:** We searched the PubMed, Scopus, and Web of Science databases for cross-sectional, case–control, and cohort studies focusing on musculoskeletal sequelae in patients with COVID-19 infection published between 2020 and 2025. Study quality and risk of bias were assessed using the MINORS and the ROBINS-E scales, respectively. **Results:** Thirteen studies (n = 5657 patients) met the eligibility criteria. Seventy-six percent of studies indicated muscle weakness as the most common sequela, primarily in older adults and individuals with comorbidities (obesity, diabetes, and chronic obstructive pulmonary disease). General fatigue (reported in 76% of the studies) significantly influenced patients’ daily lives, whereas 90% of patients reported some level of deterioration in their QoL, primarily regarding mental health, bodily pain, and physical performance. **Conclusions:** Patients with PCS who required mechanical ventilation showed reduced muscle strength and poor physical performance, especially older adults. Inactive individuals had worse musculoskeletal sequelae, while physical activity was associated with better strength levels. Although QoL improved after 12 months, the combination of aerobic exercise with adequate nutrition is essential to promote muscle recovery, reduce fatigue, and improve overall functional capacity in post-COVID-19 patients.

## 1. Introduction

The coronavirus outbreak in 2019 (caused by severe acute respiratory syndrome coronavirus 2 (SARS-CoV-2) originated in China and was labeled as COVID-19 by the World Health Organization (WHO). The coronavirus infection manifests as severe acute respiratory syndrome, accompanied by fever, cough, and dyspnea, along with direct and indirect effects on multiple organ systems, including the musculoskeletal system [1].

In March 2025, the WHO published a report stating that 777.6 million cases and 7.1 million deaths due to COVID-19 have been recorded around the world [2]. The Global Burden of Disease in Latin American and Caribbean countries highlighted that during 2020–2021, the countries Bolivia, Perú, Nicaragua, Haiti, and Paraguay had the highest rates of disability-adjusted life years; premature mortality was noted as a principal marker of disease burden, while male sex, poverty, and older age were identified as the main risk factors [3]. In Colombia, the Ministry of Health reported approximately 6.3 million cases of COVID-19 infection in March 2025 with a mortality rate of 2.2% (142,780 fatal cases) [4].

Several studies conducted on infected patients have shown that, after the acute phase of infection, a variety of sequelae, such as cardiac, muscular, articular, respiratory, nervous, and psychological effects, may appear, affecting several organ systems and tissues [5,6].

Muscle function impairments in individuals with long COVID can persist up to 12 months post-discharge, though some studies report gradual improvement [7,8,9]; the variability in outcomes primarily stems from differences in disease severity, age, and comorbidities. Patients recovering from severe COVID-19 often exhibit worse physical performance compared to those with milder illness. Additionally, comorbid conditions, including obesity, pulmonary disease, and low muscle mass, are reported to be strongly associated with prolonged functional limitations, emphasizing their importance in future long COVID research [10,11,12].

Previous cohort studies have determined that long COVID is associated with a marked deterioration in the patient’s health-related quality of life (HRQoL), with persistent fatigue identified as one of the most prevalent symptoms, which significantly compromises physical functioning and overall HRQoL. Notably, higher self-reported fatigue levels correlate with greater reductions in HRQoL metrics; however, when statistical models adjusted for fatigue, the estimated impact of long COVID on HRQoL diminished, indicating that fatigue serves as a critical mediator in the pathway linking post-viral sequelae and impaired functional outcomes. These findings underscore the importance of targeted physiotherapeutic interventions aimed at reducing fatigue to improve recovery trajectories in individuals with long COVID [13,14].

Post-COVID Syndrome (PCS), also called long COVID, chronic COVID, or post-COVID condition, has been defined by the National Academies of Science, Engineering, and Medicine as a chronic condition that occurs after SARS-CoV-2 infection. The condition manifests at least three months after the initial infection and presents as a persistent, relapsing, remitting, or progressively worsening disease affecting one or more organ systems. Risk factors for PCS include female sex, recurrent infections, and severe initial infections, while common symptoms include persistent migraines, arrhythmias, dysautonomia, chronic fatigue syndrome, and hypoxemia [15]. Typical musculoskeletal sequelae of PCS include fatigue, myalgias, arthralgias, and sarcopenia, which can limit daily activities in affected patients, resulting in significant disability [16].

Recent Cochrane and WHO-endorsed analyses highlight fatigue, dyspnea, muscle weakness, and joint pain as the most prevalent musculoskeletal sequelae of Post-COVID-19 Syndrome (PCS), affecting an estimated 65 million individuals worldwide. Rehabilitation strategies focused on exercise, respiratory, and strengthening interventions—particularly comprehensive pulmonary and multicomponent telerehabilitation programs—have demonstrated modest yet consistent improvements in fatigue, exercise tolerance, and quality of life, although the certainty of evidence remains low due to methodological heterogeneity [17]. Complementary evidence from other chronic conditions supports the use of aerobic and resistance training, as well as educational and self-management interventions, to counteract post-viral fatigue and physical deconditioning [18]. Furthermore, both WHO and Cochrane Rehabilitation stress that early, multidisciplinary rehabilitation programs can mitigate functional decline and foster the recovery of physical capacity and psychosocial well-being [19].

This systematic review aims to synthesize current evidence on the musculoskeletal sequelae of PCS, with particular emphasis on peripheral muscle strength, fatigue, and health-related quality of life as reported in studies published between 2020 and 2025. It also explores the influence of age, sex, comorbidities, and the severity of acute infection symptoms. By integrating objective functional assessments with patient-reported outcomes, this review seeks to elucidate the magnitude and clinical significance of these impairments and to inform evidence-based rehabilitation strategies that enhance recovery, functional performance, and long-term well-being among individuals with PCS.

## 2. Methods

Following the Preferred Reporting Items for Systematic reviews and Meta-Analyses (PRISMA) guidelines (2020) for conducting this systematic review. The review protocol was retrospectively registered at Open Science Framework https://doi.org/10.17605/OSF.IO/AE4JK (accessed on 18 November 2024).

### 2.1. Eligibility Criteria

A literature review was conducted between 1 August 2023 and 31 July 2024 to extract studies focusing on musculoskeletal sequelae in patients with COVID-19 infection. Publications from 2020 and 2025 in Spanish, English, or Portuguese were included, as these represent the predominant languages of scientific dissemination in the Americas and Europe on Post-COVID-19 research. These encompassed clinical trials, case–control studies, case reports, case series with over 30 patients, and cohort studies published in indexed journals that addressed the population, intervention/exposure, comparison, and outcomes (PICO) questions listed in Table 1. The PICO framework describes the population, intervention/exposure, comparison, and outcomes. Patients included in the studies must have been followed for at least 12 weeks after infection, as confirmed by polymerase chain reaction (PCR) documented in medical records. The outcomes examined in the present review included physical function, fatigue, and muscle strength, validated through a measurement scale or battery of tests.

### 2.2. Exclusion Criteria

Studies were excluded if the measurements did not specifically correspond to the outcomes of interest. Additionally, studies were excluded if the population did not meet the specified follow-up duration. Case reports and small series with fewer than 30 participants were excluded to maintain methodological rigor and ensure sufficient statistical power and external validity.

### 2.3. Data Sources and Search Strategies

A comprehensive literature search was conducted across eight electronic databases: Web of Science, PubMed, Scopus, ScienceDirect, Lilacs, Springer, PeDRo, and SciELO. The search strategy followed PRISMA 2020 recommendations and was designed to ensure the exhaustive identification of studies addressing musculoskeletal sequelae in patients with post-COVID-19 syndrome (PCS). Boolean operators “AND” and “OR” were used to combine terms related to the exposure (Long COVID-19, post-acute COVID-19 syndrome [PACS], or persistent COVID-19) and outcomes (physical therapy, physiotherapy, fatigue, muscle weakness, sarcopenia, and functional status).

The following search strings were applied: (“Post-COVID-19 Syndrome” OR “Long COVID” OR “Chronic COVID Syndrome”) AND “Functional Status”; (“Post-COVID-19 Syndrome” OR “Long COVID” OR “Chronic COVID Syndrome”) AND “Muscle Strength”; (“Post-COVID-19 Syndrome” OR “Long COVID” OR “Chronic COVID Syndrome”) AND “Sarcopenia”; (“Post-COVID-19 Syndrome” OR “Long COVID” OR “Chronic COVID Syndrome”) AND “Fatigue”; (“Post-COVID-19 Syndrome” OR “Long COVID” OR “Chronic COVID Syndrome”) AND (“Musculoskeletal System” AND “Fatigue”); and (“Post-COVID-19 Syndrome” OR “Long COVID” OR “Chronic COVID Syndrome”) AND (“Musculoskeletal System” AND “Weakness”).

All retrieved studies were imported into EndNote 2024 reference management software for duplicate removal and further screening. No date restrictions were applied, and searches included studies published in English, Portuguese, or Spanish.

### 2.4. Study Selection

The articles were analyzed to determine the relationship between the obtained results and the specific objectives outlined in this review. That is, musculoskeletal sequelae validated through a measurement scale or battery of tests, correlation with the patient’s age and sex, and sequelae related to functionality.

### 2.5. Data Collection Process

The search was conducted in electronic databases using the aforementioned criteria. Two independent researchers performed an initial screening of the study titles and abstracts after searching various databases. Each reviewer produced a list of studies, which were then reviewed by both reviewers until consensus for inclusion was reached. A third reviewer, who was blinded to the reviewers’ responses, was consulted in case of disagreement. An article was included if there was agreement between the reviewers; if there was no agreement, a fourth evaluator made the final decision. In the next step, full texts of the selected studies were reviewed according to the predetermined eligibility criteria (inclusion and exclusion). Any disagreements regarding eligibility, quality, or data extraction were resolved through consensus with the other reviewer.

Both reviewers independently extracted the following data from the included studies using a pre-designed MS Excel template: author and year, study type, country, sample size, age characteristics, comorbidities of the population, musculoskeletal sequelae, presence of fatigue, HRQoL, functional independence, and the measurement scale used for each sequela.

### 2.6. Quality Assessment

The quality of the included studies was assessed independently and blinded by both reviewers using the MINORS scale. This scale evaluates the following aspects for non-comparative studies: clarity of the objective, patient inclusion, prospective data collection, adequacy of outcome reporting relative to the study objective, impartiality of outcome assessment, adequacy of the follow-up period for the study objective, loss to follow-up below 5%, and sample size estimation, among others. For comparative studies, additional criteria include adequacy of the control group, concurrent management of groups, baseline group equivalence, and adequacy of statistical analysis. Each of these items was scored on a scale from 0 to 2, where 0 indicates not reported, 1 indicates reported but inadequately, and 2 indicates reported adequately. An ideal total score (sum of points across the 8 domains) of ≥12 for comparative studies (total = 24 points) and ≥8 for non-comparative studies (total = 16 points) was considered representative of a good quality study [20].

### 2.7. Risk of Bias

All relevant information from the included studies was compiled and analyzed using the Review Manager tool (version 5.4.1) developed by the Cochrane Collaboration. The two reviewers independently assessed the risk of bias using the ROBINS-E scale [21]. The scale evaluates the following aspects regarding possible bias: random sequence generation, allocation concealment (selection bias), blinding of participants and personnel (performance bias), blinding of outcome assessment (detection bias), losses during follow-up (attrition bias), and selective reporting (reporting bias). All criteria adhered to the ROBINS-E scale standards.

For each domain, ROBINS-E uses a series of “signaling questions” to guide the assessment of risk of bias. The answers to these questions are used to make three judgments: risk of bias in the outcome (determining whether the risk of bias in that specific domain is low, moderate, serious, or critical); direction of bias (assessing whether the bias could be overestimating or underestimating the effect of the exposure); and threat to the conclusions (assessing whether the risk of bias is high enough to threaten conclusions about the effect of the exposure). After completing the seven bias domains, an overall judgment is made for each of these three considerations.

## 3. Results

**Figure 1:**
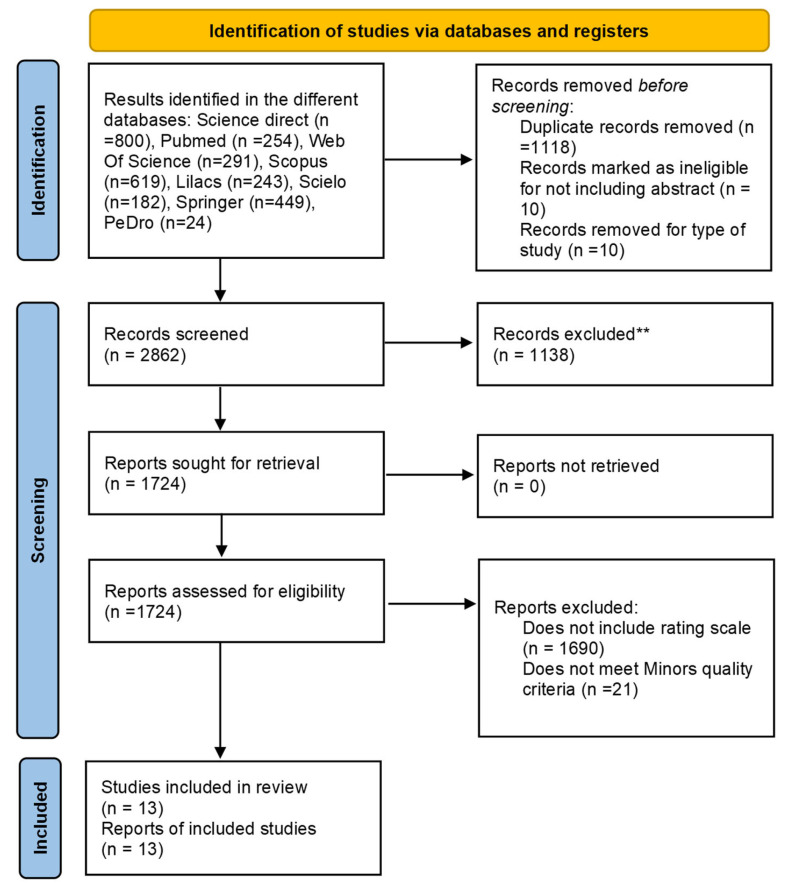
PRISMA flow chart for study selection. ** A total of 1118 records were excluded using EndNote 2024.

A comprehensive search for scientific evidence was conducted across multiple databases, including Web of Science (291 results), PubMed (254), Scopus (619), ScienceDirect (800), LILACS (243), Springer (449), PEDro (24), and SciELO (182). The application of predefined search strategies across these sources yielded a total of 2862 articles. All retrieved studies were imported into EndNote for reference management, and duplicates were identified and removed. After screening titles and abstracts, 1724 articles were selected for full-text review, of which 1690 were excluded for not including a standardized rating or outcome scale. Subsequent methodological quality appraisal using the MINORS instrument led to the exclusion of an additional 21 studies, primarily due to insufficient reporting or unclear information regarding follow-up adequacy in relation to post-COVID-19 timelines, participant attrition (<5%), sample size calculation, and the selection of appropriate control or comparison groups. Finally, 13 studies were included in the quality synthesis after excluding duplicates and articles that did not meet the inclusion or quality criteria. Figure 1 and Table 2 illustrate the study selection process and the characteristics of the included studies.

### 3.1. Characteristics of the Included Studies

The 13 studies included in the analysis comprised a total of 5657 patients with a history of COVID-19 infection. After at least 12 weeks from diagnosis, these patients continued to experience symptoms such as fatigue, difficulty concentrating, memory changes, recurrent headaches, and sleep problems, among others. More than half (56%) of the 5657 patients were male, aged between 33 and 70 years. Most patients had been hospitalized, requiring oxygen support either in the intensive care unit (ICU)—where a significant percentage required mechanical ventilation—or in general wards, with a median hospital stay of 15.23 days [22,23,24,25,26,27,28,29]. Males were more affected by severe COVID-19, requiring this level of oxygen support [22,23,24,25,26,27,28,29,30,31,32]; however, in three studies, the sample population did not require hospitalization [26,33,34]. All measurements were conducted at least three months after the COVID-19 diagnosis. Sociodemographic and clinical variables were primarily extracted from medical records, while assessments of muscle strength, fatigue, and QoL were performed at a single time point [22,23,24,25,26,27,28,29,30,31,32,33,34]. Two studies followed up the population with persistent symptoms for more than 12 months [23,32].

**Table 2:** Characteristics of the studies.

Authors/Year	Country	Sample/ %PCC	Clinical Data	Time of Hospitalization in Days	Sex		$\mathrm{Age}\;\bar{x}$ and S	Type of Study	Sequelae Assessed	Outcome Assessment Methods
					M	F				
Battistella 2022 [22]	Brazil	801/ 100% PCC	No O_2_ support (10%), O_2_ support (48.1%), intubation (41.5%)	Median of 15.2 (IQR 10.3; 21.6)	52.5%	47.4%	55.35 ± 14.58	Cohorts	Muscle strength, fatigue, HRQoL	Performed on average x = 6.56 (S = 1.58) months after hospital discharge, through a series of tests included in a hospital protocol
De Azevedo Vieira 2023 [23]	Brazil	350/61% PCC	Previous hospitalization (41.7%), Admitted in ICU (21.7%), non-hospitalization (36.6%).	NR	38.9%	61.1%	55.5	Longitudinal observational	Muscle strength, fatigue, HRQoL	Patients received telemonitoring for 12 weeks, followed by clinical reassessment at a median of 96 days. Persistent symptoms prompted evaluations at six, nine, and twelve months during follow-up.
Galluzzo 2023 [24]	Italy	1846/ 100% PCC	Previous hospitalization no O_2_ support (11%), Previous hospitalization O_2_ support (21%), Previous hospitalization NIV, CPAP (19%), Admitted in ICU (6%), non-hospitalization (43%).	Median of 18.9 (IQR 18.6; 19.3)	53%	47%	55.2 ± 14.4	Cohorts	Muscle strength	Participants reported physical activity levels pre- and post-COVID-19, including resistance training (≥150 min/week over 3 months). They were categorized as inactive, formerly active, or consistently active based on their activity patterns.
Ghosn 2023 [25]	France	737/ 100% PCC	Previous hospitalization O_2_ support (65%), Previous hospitalization NIV, CPAP (17%), Admitted in ICU (32.8%). In patients with O_2_ support, are included admitted and not admitted to the ICU.	Median of 9 (IQR 5; 17)	65%	35%	61 ± 8.3	Cohorts	Muscle Strength, HRQoL	Follow-up was planned with a physician’s visit at month 3, month 6 and month 12 after hospital admission. At the M12-visit, a measure of the functional independence, muscle strength of each limb, health-related quality of life and on their psychological distress.
Coscia 2023 [33]	Italy	506/ 100% PCC	100% non-hospitalized. 88% are physically active, 12% sedentary people.	0	73%	27%	33 ± 14.4	Cases and controls	Fatigue	The study included active individuals with varying aerobic and anaerobic activity levels who completed a stress test pre-infection, grouped as cross-country athletes, mountain amateurs, ski instructors, or sedentary controls.
De Castro 2024 [26]	Brazil	227/66% PCC	100% required hospitalization. 33% without decreased lung function; 43% without decreased lung function but with fatigue; 26% with decreased lung function and fatigue	Median of 12.8 (IQR 5; 18.5)	45%	55%	52 ± 15.2	Cases and Controls. Observational cross-sectional	Muscle strength	After ≅6 to 12 months (median of 202 days) of hospital discharge, patients who were hospitalized in 2020, during the first wave were invited to follow-up assessment. Data from the hospitalization period were obtained from the medical records of each volunteer.
De Oliveira 2022 [34]	Brazil	37/ 100% PCC	100% non-hospitalized	0	0	100%	52.9 ± 12.8	Observational cross-sectional	Muscle strength, fatigue, HRQoL	The tests were applied 8.1 ± 3.2 months after the diagnosis of COVID-19, in physically active women according to the IPAQ test.
Gunnarsson 2023 [27]	Denmark	292/ 100% PCC	50% non-hospitalized; 50% hospitalized	Median 8 (IQR 5–14)	43.8%	56.2%	51.9 ± 15.2	Observational cross-sectional	Muscle strength	Patients at the post-COVID-19 clinic underwent physical, cognitive, and physician evaluations. Beforehand, they completed nurse-conducted telephone questionnaires, with assessments occurring 217.2 ± 111.5 days post-infection diagnosis.
Stavrou 2022 [30]	Greece	60/66% PCC	50% hospitalized; 50% without history of or active COVID-19 but with obstructive sleep Apnea Syndrome	NR	83%	17%	51.7 ± 6.5	Observational cross-sectional	Muscle strength, HRQoL	The tests were carried out in the same laboratory, between 9:30 a.m. and 1:30 p.m., after the evaluation of anthropometric characteristics and body composition, with controlled temperature and humidity.
De Lorenzo 2022 [31]	Italy	97/ 100% PCC	100% COVID-19 survivors hospitalized during the first pandemic wave who underwent a CT scan and had baseline and 6-month clinical and anthropometric data available for analyses	Median 16.5 (9.9; 28.4)	79.5%	20.5%	60 ± 8.4	Observational cross-sectional	Muscle mass HRQoL	The 6-month follow-up included an internal medicine assessment, anthropometric measurements, and a CT scan conducted during hospitalization. Follow-up occurred 184.8 days (IQR 176.5–192) post-discharge.
Do Amaral 2024 [28]	Brazil	113/ 100% PCC	6.7% ICU on admission, 5.3% invasive mechanical ventilation on admission, 93.3% Hospitalized with oxygen support on admission	NR	46%	54%	48 ± 12.8	Longitudinal observational	Muscle strength, HRQoL	COVID-19 patients were followed up after hospitalization and performed pulmonary function and physical capacity tests 120 days after discharge. Muscle strength was evaluated with dynamometry on day 1 of admission to hospitalization and on day 120 after hospital discharge.
Martone 2022 [29]	Italy	541/ 100% PCC	39% home, 19% hospitalized-no O_2_ support, 27% hospitalized-O_2_ support. 10% Hospital NIV or CPAP, 5% invasive ventilation	Median 16.3 (IQR 14.9; 21.4)	49%	51%	53.1 ± 15.2	Observational cross-sectional	Muscle strength	Follow-up visits occurred at least 3 months after COVID-19 onset, offering comprehensive medical assessments, including detailed histories and physical exams. Muscle strength was independently assessed by a physiotherapist unaffiliated with the project.
Silva, 2024 [32]	Brazil	50/84% PCC	100% hospitalized, without the need for admission to an intensive care unit.	NR	100%	0	52 ± 10.6	Observational cross-sectional	Muscle strength, HRQoL	There is an important recovery of functional capacity, with less than one-third of population showing an abnormal handgrip strenght and quadriceps strength. After three years of COVID-19 hospitalization only one-third of patients have mechanical and/or diffusion pulmonary changes. However, most of them maintain some damage in muscle strength, and QoL remains deteriorated.

PCC: post-COVID-19 condition; M: male; F: female; $\bar{x}$: arithmetic mean; S: standard deviation; ICU: Intensive Care Unit; HRQoL: health-related quality of life; NIV: Non-Invasive Ventilation; CPAP: continuous positive airway pressure; IPAQ: e International Physical Activity questionary; CT: computed tomography. IQR: Interquartile range.

The included studies provided relevant data on muscle strength evaluation, the presence of fatigue and muscle weakness, and HRQoL. Muscle strength was the most frequently reported outcome in the articles [22,23,24,26,27,28,29,30,31,32,34]. De Lorenzo et al. reported skeletal muscle radiodensity using computed tomography of the paraspinal muscles. This measurement is considered the most reliable biomarker for myosteatosis, which referes to the amount of fat and fibrous tissue infiltration in the muscle. Myosteatosis is a key indicator for assessing the loss of muscle quality, representing the skeletal muscle’s ability to perform its functions effectively, and is independently associated with higher patient mortality [31].

### 3.2. Quality Assessment

The methodological quality of 21 studies was evaluated using the MINORS scale for comparative and non-comparative studies. Among these, 13 studies (62%) exceeded the threshold established to be considered good methodological quality. The main reasons for exclusion were: loss to follow-up below 5% (adequately reported in 47% of the studies) [3,12,13,14,15,35,36,37,38,39]; sample size calculation (adequately reported in 30% of studies) [40,41,42,43,44,45]; appropriate use of a control group (adequately reported in 78% of studies) [12,13,14,15,38,40,41,42,43]; concurrent management of comparison groups (adequately reported in 78% of studies) [13,15,38,41]; and equivalence of the comparison groups (adequately reported in 72% of studies) [12,13,15,38,40,41,42,43]. Items adequately reported by more than 95% of the articles were not included in this review. Among the 13 studies included in the review, only three studies (23%) inadequately reported sample size calculation, while all other criteria were adequately reported across all articles (Table 3).

### 3.3. Risk of Bias

**Figure 2:**
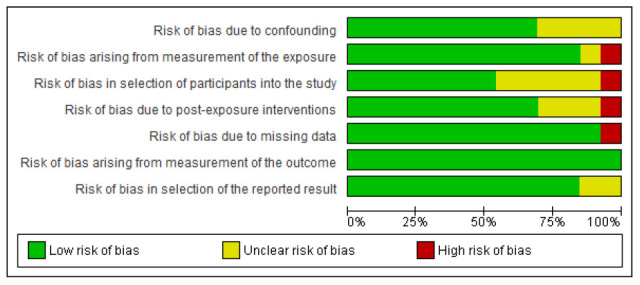
Risk of bias assessment.

**Figure 3:**
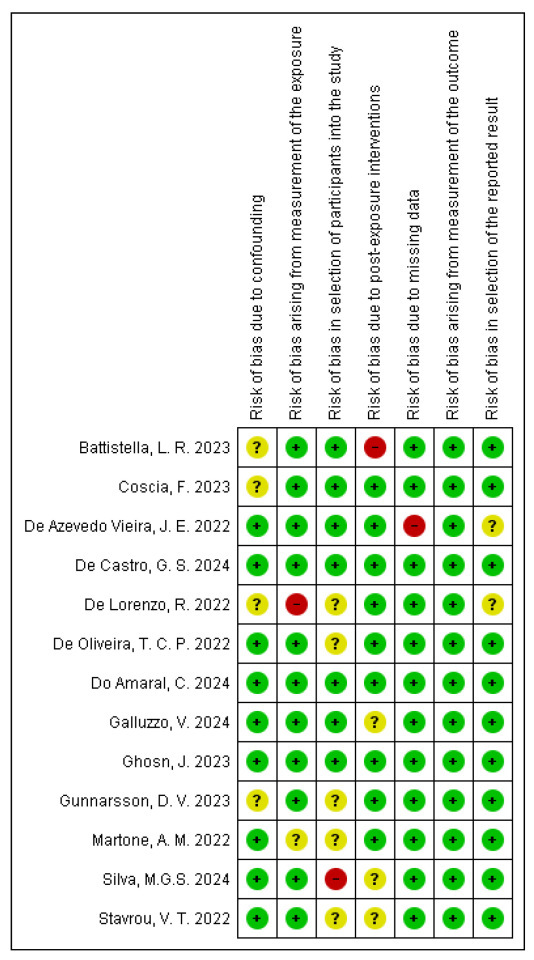
Summary of risk of bias [22,23,24,25,26,27,28,29,30,31,32,33,34]. The symbol ‘+’ denotes a low risk of bias, ‘?’ denotes an unclear risk of bias, and ‘−’ denotes a high risk of bias.

The overall risk of bias in the included studies was generally low across most evaluated domains, reflecting high methodological quality (Figure 2 and Figure 3). However, certain areas, such as confounding factors, post-COVID-19 interventions, participant loss during follow-up, and representative sampling, showed a significant proportion of unclear risk, and the findings should, therefore, be interpreted with caution. Four studies (31%) presented an unclear risk of confounding bias. Regarding exposure measurement, one study (8%) showed an unclear risk, and another study (8%) showed a high risk. For participant selection, six studies (46%) had an unclear risk. Bias due to post-exposure interventions was unclear in two studies (15%), while one study (8%) showed a high risk. Two studies (15%) presented a high risk of bias concerning missing data. Outcome measurement was low risk in all studies, whereas two studies (15%) revealed an unclear risk in selective outcome reporting. The few studies identified as having unclear or high risk of bias provided complementary insights that broaden the understanding of the phenomenon, although their findings were interpreted with analytical caution. Their inclusion enhanced the contextual and population heterogeneity of the synthesis, contributing to a more representative evidence base and highlighting priority areas for future research employing more robust designs and improved control of confounding factors.

In general, the included studies present a low frequency of information and selection biases. It should be noted that the heterogeneity and variability in the study designs primarily arise from the evolving knowledge of the natural history of this new health condition related to the recent COVID-19 pandemic. Continuing research will allow for a reduction in these biases through more precise scales and measurement instruments specific to this disease or syndrome.

### 3.4. Characteristics of Comorbidities in Patients with PCS

Among the post-COVID-19 population in the studies, as indicated in Table 4, ten studies reported that 44.16% of patients with PCS had hypertension (HTN), while another ten studies reported 28.15% type 2 diabetes mellitus (T2DM). Likewise, coronary artery disease was reported in seven studies (9.35% of patients), chronic kidney disease in four studies (7.67% of patients), and chronic obstructive pulmonary disease (COPD) was reported in eight studies (6.73% of patients). The body mass index of these patients ranged from 18 kg/m^2^ to 39.5 kg/m^2^, with a mode value of 28.36 kg/m^2^. It should be noted that individual patients could have had multiple comorbidities, as all studies considered each reported comorbidity [22,23,24,25,26,28,29,31,32,33,34].

### 3.5. Peripheral Muscle Strength Sequelae in Patients with PCS

One of the most common symptoms among patients with PCS is peripheral muscle weakness [28]. Muscle strength was primarily measured using dynamometry, as the most affected population was generally older adults. Muscle strength is a key criterion for determining sarcopenia. The European Working Group on Sarcopenia in Older People (EWGSOP2) recommends using a dynamometer to measure muscle strength when defining sarcopenia. Subjects over 65 years of age are considered to have probable sarcopenia when handgrip strength is <27 kg in males and <16 kg in females [29,30]. Dynapenia is defined as handgrip strength <30 kg for males and <20 kg for females [28]. For individuals under 65, standardized values are used [20,21,22,26,29,33]. The One-Minute Sit-to-Stand Test (1MSTST), a tool used for strength measurement, is another widely used tool due to its validity and sensitivity. For individuals under 65, standardized values based on age and gender are applied [46]. Likewise, for older adults, the Short Physical Performance Battery is often used to assess muscle strength, as it also evaluates fall risk and dependency in this age group [20,21,22,26,29,33]. Respiratory muscle strength was not assessed in the present analysis.

Seventy-six percent of the articles included in this review reported muscle strength outcomes, all documenting decreased muscle strength, as indicated in Table 5 [20,22,24,26,30,31,33]. The loss of strength was greater in older adults or patients with comorbidities such as obesity [22,24,26,30,33], T2DM, and COPD [23,24,27,28,29,31,34], as well as in patients with severe COVID-19, particularly those who required prolonged hospitalization in the ICU with intubation and sedation [22,23,24,25,28,29].

### 3.6. Muscle Fatigue in Patients with PCS

Fatigue is another commonly documented musculoskeletal sequela, described by patients as a feeling of tiredness that limits or prevents the performance of daily activities. All 13 studies (100%) included a survey asking patients about symptoms, among which fatigue was consistently reported as one of the most frequent complaints, as shown in Table 6 [22,23,24,25,26,27,28,29,30,31,32,33,34]. However, only 46% of the studies used a formal instrument to assess the physical component of fatigue. The most frequently employed tool was the Post-COVID-19 Functional Status Scale (PCFS), which evaluates the degree of limitation in performing essential daily activities during post-infection follow-up. The scale grades functional limitations as follows: grade 0 (no functional limitations), grade 1 (negligible functional limitations), grade 2 (slight functional limitations), grade 3 (moderate functional limitations), and grade 4 (severe functional limitations) [47]. Another commonly used tool is the Functional Assessment of Chronic Illness Therapy-Fatigue (FACIT-F) scale, which consists of 13 items assessing fatigue during activities of daily living (ADL), with scores ranging from 0 to 52, where lower scores indicate a lower level of fatigue [23]. The Rating of Fatigue (ROF) scale assesses fatigue during ADL using 12 descriptors accompanied by diagrams, with lower scores indicating a lower level of fatigue [48]. Both FACIT-F and ROF are applied to evaluate fatigue associated with chronic illness. Finally, the TGlittre-ADL test measures the time spent performing several tasks while carrying a backpack and walking a 10 m circuit, from the start to finish [32,34].

Regardless of the scale used, patients consistently reported limitations in performing ADLs, although only a small percentage experienced severe limitations [22,23,33,34]. Similarly, in sedentary patients or those with pre-existing lung pathology prior to COVID-19 infection, fatigue perception persisted longer compared to those who had engaged in any form of physical activity before the onset of COVID-19 [22,33]. No association was found between comorbidities such as hypertension, T2DM, or kidney pathology and fatigue perception [22,23,33,34].

### 3.7. Quality of Life-Related Sequelae in Patients with PCS

HRQoL reflects the patient’s perception of their condition, including its impact on their muscle strength and ability to perform daily activities. This perception also considers factors such as pain, anxiety, depression, and emotional well-being. All 13 articles reviewed included a survey in which patients were asked about changes in their QoL due to COVID-19 over the past month, as shown in Table 7. A significant proportion of patients reported a decline, particularly in mental health and muscle pain [22,23,24,25,26,27,28,29,30,31,32,33,34]. However, it was measured using an instrument in only 38% of the cases. The tools employed included the short-form (SF)-36 and its short version, the SF-12, both commonly used across various diseases, as well as the EQ-5D-5L and 5D-5Q [23,24,25,27,28]. Another instrument used for QoL is the Saint George Respiratory Questionnaire, which evaluates three domains related to respiratory function: symptoms, activities that cause dyspnea, and interference with ADLs [32].

Overall, the results indicate that less than 10% of the study population perceived their QoL as poor. However, the remaining 90% reported some level of deterioration, with physical performance, bodily pain, and mental health being the most significantly affected areas [22,23,25,31,34].

## 4. Discussion

This systematic review aimed to identify musculoskeletal sequelae in patients with Post-COVID-19 Syndrome (PCS). Based on the analysis of 13 selected studies, a total of 5390 patients were included. At least 12 weeks after infection was confirmed by PCR test, the studies identified common musculoskeletal sequelae of PCS, including decreased muscle strength, fatigue affecting physical function, musculoskeletal pain, and a decline in QoL, particularly in physical function and role performance [22,23,24,25,26,27,28,29,30,31,32,33,34].

Patients who required ventilatory support showed poor performance, with handgrip strength worse outcomes noted for older adults and female patients, with longer walking times in the Timed Up and Go test. However, although fatigue was present in the evaluated patients, no causal link was established between fatigue, dyspnea, muscle weakness, and overall performance in patients with PACS.

These sequelae persisted for at least 12 weeks, with notable improvements observed between 3 and 11 months after hospital discharge [24]. Furthermore, patients who were inactive prior to the pandemic or reduced their physical activity during the pandemic and had comorbidities like HTN, T2DM, and COPD experienced longer hospital stays compared to those without comorbidities; as expected, these habits negatively influenced their muscle strength, fatigue levels, and dyspnea [23,49,50].

Recent studies reveal that between 6 months and up to 2 years after infection, both hospitalized and non-hospitalized patients during the acute phase of the disease frequently experience general fatigue, dyspnea, pain, and memory loss; these symptoms tend to worsen with increased comorbidities and severity of symptoms during the acute stage. While dyspnea is initially more prevalent than fatigue, especially in hospitalized patients, it decreases significantly post-infection; however, fatigue does not follow the same pattern and persists more frequently, becoming more common than dyspnea up to 2 years after infection [50,51,52]. These findings are in line with those of Battistella, who analyzed patients who were hospitalized during the acute phase of COVID-19 infection and reported that 64.65% experienced dyspnea, while 39.18% reported fatigue between 3 and 11 months post-infection [22]. De Azevedo et al. indicated that 12 months after contracting infection, 18.8% of adults who were previously active continued to experience general fatigue, and 15.6% experienced dyspnea [23]. Coscia et al. noted that fatigue and dyspnea significantly decreased between 6 and 12 months post-infection, with a more pronounced reduction in dyspnea. They also found that individuals who engaged in regular physical activity experienced a greater reduction in symptoms compared to the sedentary population, in whom symptom improvement was less substantial [33].

This is further corroborated by the International Physical Activity Questionnaire criteria, which state that musculoskeletal sequelae are more pronounced in individuals classified as physiologically inactive [23,53,54,55]. Those who were active before and during the pandemic demonstrated superior prehensile strength, performance on the 1MSTS, and results on the 6 min walk test (6MWT). Specifically, active participants walked an average of 32 m farther than their inactive counterparts, underscoring the role of physical activity as an indicator of protection against frailty and a means of preserving independence in later life [25,34,53,56,57].

Dynapenia is defined as a decrease in muscle strength (standard values established as <30 kg-force (kgf) for males and <20 kgf for females in dynamometry assessments); it correlates with all-cause mortality, reduced functional health, decreased bone density, and depression [58]. Do Amaral studied this association and found that some patients with PACS who exhibited dynapenia also had lower scores on pulmonary function tests and reduced respiratory strength, which negatively affected their performance on the 6MWT and fatigue assessments [28,37]. A recent study revealed that 12 months post-infection, 38% of patients experienced muscle atrophy, and 56% of those with prolonged hospital stays and fatigue had not fully regained their muscle fibers. Histopathological examinations among these patients identified a loss of cytochrome C oxidase activity, other mitochondrial changes, and alterations in muscle fibers and capillary lamina, which may contribute to fatigue, muscle weakness, and dynapenia [37,55,57,59].

De Lorenzo et al. evaluated myosteatosis in post-COVID-19 patients, they assessed the quantity of muscle mass and fat in the longissimus thoracis, thoracic spinal muscles, and iliocostal lumbar muscles. They found that patients with low paravertebral muscle mass exhibited dyspnea and significant functional decline compared to those without this condition [31]. Furthermore, these patients also experienced obesity, prolonged hospital stays, and a need for ventilatory support in the ICU, indicating that myosteatosis—fat infiltration into muscle fibers negatively impacts muscle strength and QoL [31,53]. Other studies have also reported a strong association between low trunk muscle mass and reduced functional capacity in outpatient and hospitalized patients [60,61,62].

Regarding sarcopenia, as defined by the EWGSOP2 [29,30,63]. Martone evaluated its association with the persistence of COVID-19 symptoms and found that 19.5% of patients presented sarcopenia, with a higher prevalence among women [29]. This finding is significant because sarcopenia is closely linked to frailty, falls, reduced mobility, physical dependency, and mortality in older adults [37,63,64,65]. Patients with sarcopenia also showed a higher prevalence of HTN, T2DM, COPD, dyspnea, and fatigue compared to those without sarcopenia, suggesting the influence of systemic inflammation mediated by COVID-19. The findings may be related to an ongoing inflammatory state. Several studies have proposed the role of leucine (a biological substrate) as a factor contributing to fatigue and dyspnea due to weakness in skeletal and respiratory muscles, respectively [29,66,67,68]. Furthermore, studies have detected the presence of angiotensin-converting enzyme 2 in muscle tissue in COVID-19 patients. At this stage, elevated angiotensin I and II levels can exacerbate proteolysis, impair muscle regeneration, and increase muscle apoptosis, which, combined with malnutrition, physical inactivity during hospitalization, and a state of multiorgan inflammation, worsens sarcopenia. This inflammatory cascade is mediated by excessive cytokine release—including IL-1, IL-6, IL-7, IL-8, IL-9, IL-10, TNF-α, and interferons—further amplifying catabolic pathways [26,30,33,34,69].

Protein deficiency represents a critical underlying factor that accelerates this cascade by limiting substrate availability for muscle protein synthesis and mitochondrial recovery. Reduced protein intake during and after hospitalization, together with immobilization and sustained inflammatory activation, markedly decreases lean mass and muscle quality [29]. These changes not only impair contractile function but also predispose patients to dynapenia. In COVID-19 survivors, low serum albumin and poor nutritional status have been directly associated with slower improvement in grip strength and persistent fatigue, dyspnea, and exercise intolerance, all of which contribute to a diminished quality of life [26]. In the chronic post-infection stage, inadequate protein replenishment and continuous low-grade inflammation perpetuate anabolic resistance and hinder muscle repair. De Lorenzo et al. demonstrated that myosteatosis independently predicts long-term dyspnea and mobility problems six months after COVID-19, regardless of age, sex, or body mass index [26,29,31,66,67,68].

The increased prevalence of comorbidities observed in patients with PCS may be explained by the interaction of several biological mechanisms. Persistent low-grade inflammation and immune dysregulation contribute to endothelial injury, microvascular dysfunction, and oxidative stress, which can exacerbate pre-existing metabolic and cardiovascular conditions [26]. Mitochondrial impairment, driven by cytokine-induced oxidative damage, disrupts cellular energy metabolism and promotes chronic fatigue, skeletal muscle atrophy, and insulin resistance [58]. Additionally, the overactivation of the renin-angiotensin system in skeletal muscle increases proteolysis and impairs muscle regeneration, perpetuating sarcopenia and dinapenia [29,59]. Together, these processes sustain a chronic catabolic state and systemic inflammation that not only prolong musculoskeletal impairment but also accelerate the progression of comorbidities such as type 2 diabetes, hypertension, and chronic obstructive pulmonary disease.

Regarding HRQoL, several factors, including the severity of COVID-19, age, sex, exercise capacity, and psychosocial status, play a significant role in shaping the patient’s QoL post-infection. Recent studies indicate that approximately 60% of patients report a decline in their QoL compared to their perceptions before infection, with particularly noticeable decreases in physical and mental HRQoL. Many individuals cite limitations in carrying out daily family activities; however, they do not report significant mobility issues. The primary factors contributing to the deterioration of HRQoL after COVID-19 include female sex, older age, the presence of comorbidities, ICU admission, extended ICU stay, and the need for mechanical ventilation [52,70,71,72,73,74,75,76]. Similar findings were noted in our systematic review. At 12 months post-COVID-19 diagnosis, significant improvements were observed in QoL, general fatigue, and lung function during follow-up. However, muscle strength exhibited a slower rate of recovery, particularly in those who had required hospitalization and ventilatory support during active COVID-19 illness. While HRQoL improved, it remained suboptimal after 12 months, particularly in aspects related to muscle strength [16,20]. Patients also experienced notable changes in functional capacity, physical performance, daily activities, pain, and anxiety [22,23].

After COVID-19 infection, patients admitted to an ICU experience numerous symptoms referred to as Post Intensive Care Syndrome, which occurs when patients experience new or worsening impairments in at least one of three domains: physical, cognitive, and mental and social function during ICU stay and after discharge [77]. This condition is part of chronic critical illness [72] observed in 30–50% of critically ill patients, characterized by a multiorgan response that includes persistent inflammation, immunosuppression, and catabolism, leading to recurrent infections, metabolic derangement, and muscle wasting [78,79].

The musculoskeletal sequelae observed in patients with post-COVID-19 syndrome (PCS) have profound implications for physiotherapy and long-term recovery. Decreased muscle strength, persistent fatigue, and musculoskeletal pain are principal determinants of reduced quality of life (QoL) and functional independence [23,33]. Individuals requiring ventilatory support or extended hospitalization exhibit the poorest physical outcomes, with older adults and women demonstrating slower recovery trajectories [25,32]. Physical inactivity during and after infection amplifies the risk of dynapenia and sarcopenia, predisposing patients to frailty, impaired mobility, and long-term dependence [29]. Pathophysiological mechanisms involve systemic inflammation, mitochondrial dysfunction, and activation of proteolytic pathways mediated by angiotensin II, cytokine overexpression, and hypoxia-induced catabolism [26,27,30]. Inflammation-driven muscle degradation, compounded by pre-existing comorbidities such as hypertension and type 2 diabetes mellitus, sustains weakness and functional decline despite improvements in lung function and fatigue at 6–12 months post-infection [23,26,34]. These findings highlight the biological continuum linking skeletal muscle impairment to systemic inflammation, metabolic dysregulation, and inactivity in PCS.

Recent studies have identified multiple alterations in biomarkers associated with musculoskeletal dysfunction in post–COVID-19 syndrome, indicating a multifactorial process involving systemic inflammation, protein catabolism, and dysregulation of tissue remodeling. Decreased levels of cartilage oligomeric matrix protein (COMP) and osteocalcin have been reported, suggesting cartilage degradation and impaired bone formation, while increased concentrations of hyaluronic acid, alkaline phosphatase (ALP), procollagen type I N-terminal propeptide (PINP), osteopontin, and matrix metalloproteinases (MMP-3 and MMP-9) reflect ongoing inflammation and connective-tissue breakdown. Additionally, alterations in myostatin, insulin-like growth factor 1 (IGF-1), follistatin, and creatine kinase point to significant muscle involvement, consistent with sarcopenia, weakness, and functional decline in post–COVID-19 patients. Collectively, these findings support the hypothesis that musculoskeletal dysfunction after SARS-CoV-2 infection is not solely a consequence of prolonged immobilization or critical illness, but rather results from persistent activation of inflammatory and catabolic pathways, which may represent potential therapeutic targets for early intervention and personalized rehabilitation strategies [80].

Given these mechanisms, rehabilitation assumes a central role in mitigating long-term disability. Evidence from multicentre and systematic reviews demonstrates that individualized physiotherapy—including progressive resistance and aerobic training—significantly improves exercise capacity, muscle strength, and QoL in post-acute COVID-19 survivors [19,81]. Programs must integrate gradual load progression, energy conservation strategies, and nutritional optimization, emphasizing adequate protein intake and vitamin D supplementation to counteract inflammation-induced catabolism [34,61]. Telerehabilitation and home-based interventions are effective adjuncts for patients with limited mobility or persistent fatigue, while early screening for sarcopenia using handgrip dynamometry, chair-stand, or imaging-based muscle quality assessment facilitates targeted therapy [19,29,34]. Collectively, these approaches underscore the need for precision rehabilitation models tailored to the biological and functional heterogeneity of PCS, aiming to restore physical performance, preserve independence, and enhance long-term health outcomes.

This systematic review, conducted 5 years after the onset of the pandemic, successfully gathered and analyzed information from various countries, revealing the global similarities in musculoskeletal sequelae of COVID-19. The findings primarily focus on muscle involvement and its consequences, including peripheral muscle fatigue and deterioration in QoL, and underscore the critical role of continuous physical activity throughout life as a preventive measure against the onset and progression of various pathologies. Our review adheres to the international guidelines using the PRISMA framework, ensuring methodological rigor. However, some limitations should be noted, such as the heterogeneity of the tools employed to assess muscle strength, fatigue, and QoL in the included studies, hindering direct comparisons between studies. This methodological variability—spanning scales such as PCFS, FACIT-F, ROF, TGlittre-ADL, SF-36, SF-12, EQ-5D-5L, and SGRQ—reflects the absence of a unified standard for evaluating functional and quality-of-life outcomes in Post-COVID-19 Syndrome. While this heterogeneity limits the statistical comparability and synthesis of quantitative results, it also enriches the interpretability of findings by capturing diverse aspects of functional impairment and recovery across different clinical contexts. Consequently, this diversity underscores the need for future research employing standardized, validated instruments to enhance the consistency and comparability of evidence in this emerging field. Additionally, not all studies provided detailed explanations of the underlying pathophysiology; thus the results obtained in this review should be interpreted carefully.

## 5. Conclusions

This systematic review highlights the persistent musculoskeletal sequelae following COVID-19 infection, characterized by peripheral muscle weakness, fatigue, and a sustained decline in health-related quality of life. The interplay between systemic inflammation, mitochondrial dysfunction, and malnutrition—particularly protein deficiency—contributes to anabolic resistance, myosteatosis, and sarcopenia, with women and older adults being disproportionately affected. Nutritional insufficiency during and after hospitalization exacerbates muscle catabolism and delays recovery, while the persistence of fatigue and reduced muscle strength beyond 12 months underscores the chronicity of these alterations. Physiotherapeutic rehabilitation emerges as a cornerstone of recovery, emphasizing progressive resistance and aerobic training, energy conservation, and nutritional optimization to counteract inflammation-driven muscle degradation. Early detection of sarcopenia using dynamometry or imaging, coupled with individualized rehabilitation and protein-rich nutritional strategies, is crucial to restoring functional capacity and preventing long-term dependence. Ultimately, addressing post-COVID-19 musculoskeletal sequelae demands a multidisciplinary, evidence-based approach that integrates physiological, nutritional, and psychosocial interventions to enhance physical performance, reduce fatigue, and improve quality of life in survivors. These findings emphasize the necessity of personalized physiotherapy interventions and underscore the importance of maintaining regular physical activity to mitigate long-term consequences associated with PCS. For researchers, the present study provides valuable baseline information to further explore the musculoskeletal outcomes of PCS and determine the optimal interventions.

## Figures and Tables

**Table 1:** Components of the PICO framework for study selection.

P (POPULATION)	I (INTERVENTION /EXPOSITION)	C (COMPARISON)	O (OUTCOMES)
Adult patients with symptoms related to COVID-19 (without another possible explanation) persist for more than 12 weeks. Studies including both hospitalized and non-hospitalized populations were eligible.	Exposure to SARS-CoV-2 infection confirmed by PCR, antigen, or serological testing, followed by the onset of post-COVID-19 musculoskeletal sequelae.	Comparisons were established based on sex, age, and presence of comorbidities, when reported, as well as between symptomatic and asymptomatic individuals or between varying severity levels of the acute infection.	Primary outcomes: muscle strength (handgrip dynamometry, lower limb strength). Secondary outcomes: fatigue (PCFS, FACIT-F, ROF), physical function (SF-36, SF-12, EQ-5D-5L), physical role, and overall quality of life.

**Table 3:** Quality of the papers.

Author	Year	Researcher 1	Researcher 2	Consensus
Battistella 2022 [22]	2022	16	16	16
De Azevedo Vieira 2023 [23]	2023	16	16	16
Galluzzo 2023 [24]	2023	16	16	16
Ghosn 2023 [25]	2023	16	16	16
Coscia 2023 [33]	2023	24	24	24
De Castro 2024 [26]	2024	24	24	24
De Oliveira 2022 [34]	2022	24	24	24
Gunnarsson 2023 [27]	2023	24	24	24
Stavrou 2022 [30]	2022	23	23	23
De Lorenzo 2022 [31]	2022	23	23	23
Do Amaral 2024 [28]	2024	23	23	23
Martone 2022 [29]	2022	24	24	24
Silva, 2024 [32]	2024	12	12	12

**Table 4:** Characteristics of the comorbidities in patients with post-COVID-19 syndrome (n = 5657).

Authors	Year	Characteristics of the Comorbidities Post-COVID-19 Syndrome Population.					
		HTN	T2DM	Coronary Heart Disease	BMI	Chronic Kidney Disease	COPD
Battistella 2022 [22]	2022	57.68%	36.45%	NR	NR	(31–34) NR	NR
De Azevedo Vieira 2023 [23]	2023	48%	25.10%	8.60%	^†^ 28.7 Range (25–33)	NR	6.30%
Galluzzo 2023 [24]	2023	33%	10%	2%	^†^ 26.5 Range (21.7–31.3)	2%	7%
Ghosn 2023 [25]	2023	39%	19%	16%	NR	8%	NR
Coscia 2023 [33]	2023	NR	NR	NR	^†^ 21.8 Range (18–25.5)	NR	NR
De Castro 2024 [26]	2024	78%	88%	26%	^†^ 29.9 Range (24.9–37.9)	NR	2%
De Oliveira 2022 [34]	2022	45.90%	27%	2.70%	^†^ 31.1 Range (23.4–38.5)	NR	13.50%
Gunnarsson 2023 [27]	2023	NR	NR	NR	^†^ 27.3 Range (18.3–39.1)	NR	3.20%
Stavrou 2022 [30]	2022	NR	NR	NR	^†^ 29.7 Range (25.4–39.5)	NR	NR
De Lorenzo 2022 [31]	2022	38.50%	20.30%	8.20%	^†^ 29.7 Range (24.8–33.8)	12.70%	6.50%
Do Amaral 2024 [28]	2024	34.50%	23.90%	NR	^†^ 33.2 Range (31.9–34.4)	NR	7.40%
Martone 2022 [29]	2022	29%	8%	2%	^†^ 25.7 Range (21.1–30)	8%	8%
Silva 2024 [32]	2024	38.1%	23.8%	NR	^†^ 30.1 Range (24.7–35.4)	NR	NR

BMI: body mass index; COPD: chronic obstructive pulmonary disease; HTN: Hypertension; T2DM: type 2 diabetes mellitus; NR: not reported, ^†^ average.

**Table 5:** Peripheral muscle strength sequelae in patients with post-COVID-19 syndrome (N = 5657).

Authors	Year	Strength Sequelae	Measurement Scale
Battistella 2022 [22]	2022	^†^ 19 Rep.	1MSTST
		^†^ 21.22 Kgf ± 12.70	Manual dynamometry
De Azevedo Vieira 2023 [23]	2023	^†^ 25.45 kgf ± 0.64	Manual dynamometry
Galluzzo 2023 [24]	2023	^†^ 24.8 Rep.	1MSTST
		^†^ 27.85 Kgf ± 0.44	Manual dynamometry
De Castro 2024 [26]	2024	^†^ 15.6 Rep.	1MSTST
		^†^ 23.28 kgf ± 20.96	Manual dynamometry
De Oliveira 2022 [34]	2022	^†^ 31 kgf ± 3.8	Manual dynamometry
		^†^ 24.9 kgf ± 9	Lower limb dynamometry
Gunnarsson 2023 [27]	2023	^†^ 14.3 ± 6 Rep.	30SSTST
		^†^ 33.0 ± 12	Manual dynamometry
Stavrou 2022 [30]	2022	^†^ 39.2 ± 10.3	Manual dynamometry
Do Amaral 2024 [28]	2024	^†^ 30.2 kgf ± 6.2	Manual dynamometry
Martone 2022 [29]	2022	^†^ 26.2 kgf ± 8	Manual dynamometry
		^†^ 26.2 ± 8.9 Rep.	1MSTST
Silva 2024 [32]	2024	^†^ 25.3 Kgf ± 4.1	Manual dynamometry
		^†^ 34.1 Kgf ± 1.5	Quadriceps dynamometry

1MSTST: 1-Min Sit-To-Stand Test; 30SSTST: 30-S Sit-To-Stand Test; kgf: Kilogram Force; Rep: Repetitions. ^†^ average.

**Table 6:** Presence of fatigue in patients with post-COVID-19 syndrome (n = 5657).

Authors	Year	Fatigue Sequelae	Measurement Scale
Battistella 2023 [22]	2023	70.86% of the population with limitations in ABVD, 5.62% are severe	PCFS
De Azevedo Vieira 2022 [23,33]	2022	Median of 28 (20–36)	FACIT-F
Coscia 2023 [33]	2023	It had a score of 7 at 6 months post-COVID-19; at 12 months, it decreased between 4 and 5 points for the active group and between 3.6 and 3.9 for the sedentary group	ROF
De Oliveira 2022 [34]	2022	2.7% severe functional limitation, 37.9% moderate limitation, 32.4% little limitation, 27% no limitation	PCFS
Soares 2024 [32]	2024	Total time observed (min) 3.3 (3.1–4.1) Total time predicted (min) 3 (2.7–3.4)	TGlittre-ADL Test
Gunnarsson 2023 [27]	2023	73% of patients reported a score between 2 and 3 (slight to moderate)	PCFS

PCFS: Post-COVID-19 Functional Status Scale; FACIT-F: Functional Assessment of Chronic Illness Therapy-Fatigue Scale; ROF: Rating of Fatigue; TGlittre-ADL Test: Glittre Activities of Daily Living.

**Table 7:** Sequelae in HRQoL in patients with PCS (n = 5657).

Authors	Year	Health-Related Quality of Life		Measuring Scale
Battistella 2022 [22]	2022	0	29%	EQ-5D-5L
		1	39.60%	
		2	17.00%	
		3	8.62%	
		4	5.62%	
De Azevedo Vieira 2023 [23]	2023	Physical function	50 ± 25.1	SF-36
		Physical role	30.8 ± 15.5	
		Body pain	40.1 ± 17.2	
		General health perception	45.9 ± 17.5	
		Vitality	42.3 ± 18.6	
		Social function	53 ± 23.1	
		Limitations in the emotional role	38.2 ± 24.3	
		Mental health	56 ± 17.4	
Ghosn 2023 [25]	2023	Physical component	49%	SF-12
		Mental component	31%	
De Oliveira 2022 [34]	2022	Physical function	^†^ 35 (17.5–50)	SF-36
		Physical role	^†^ 0 (0–25)	
		Body pain	^†^ 40 (20–62)	
		General health perception	^†^ 45.6 (30–70)	
		Vitality	^†^ 48.6 (22.5–70)	
		Social function	^†^ 56.21 (25–75)	
		Limitations in the emotional role	^†^ 27.7 (0–91.7)	
		Mental health	^†^ 63.3 (40–81)	
De Lorenzo 2022 [31]	2022	Mobility	40% with alteration	EQ-5D
		Self-care	20% with alteration	
		Usual activities	30% with alteration	
		Body pain	48% with presence	
		Anxiety/depression	30% with presence	
		Dyspnea	35% with presence	
Silva 2024 [32]	2024	Symptoms: Complaints of respiratory problems	Symptoms score 0.121	SGRQ
		Activity: Activities that cause dyspnea	Activity scores 0.327	
		Impacts: Interference with activities of daily living	Impacts scores 0.212 Total scores 0.266	

SF-36: SF-36 Health Questionnaire; SF-12: SF-12 QoL Questionnaire; EQ-5D-5L: EuroQoL including visual analog scale (VAS); EQ-5D: EuroQoL, measure of self-perceived health; SGRQ: Saint George Respiratory Questionnaire, ^†^ average.

## Data Availability

The original contributions presented in this study are included in the article and. Further inquiries can be directed to the corresponding author.
